# Collision Tumor Comprised of Atypical Fibroxanthoma and Invasive Squamous Cell Carcinoma: A Case Report of an Extremely Rare Entity

**DOI:** 10.7759/cureus.29324

**Published:** 2022-09-19

**Authors:** Muhammad Tahir, Kurt Knowles, Thuy Phung, Leila A Cruthirds, Spencer J Liles

**Affiliations:** 1 Pathology and Laboratory Medicine, USA (University of South Alabama) Health University Hospital, Mobile, USA; 2 Surgery, USA (University of South Alabama) Health University Hospital, Mobile, USA

**Keywords:** ­skin cancer, invasive carcinoma, atypical fibroxanthoma (afx), squamous cell carcinoma (scc), collision tumor

## Abstract

Single benign or neoplastic dermal lesions are very common. Lesions with two distinct tumors occurring as a single entity are extraordinary and known as collision tumors. In the present case, the lesion was diagnosed on biopsy, and later, wide local skin excision was performed. Excision revealed that the lesion was composed of two distinct cell populations exhibiting the characteristics of two separate neoplastic entities. The histopathology revealed a single lesion comprised of moderately differentiated squamous cell carcinoma that appeared to invade an atypical fibroxanthoma. This is unprecedented in the English literature and the first such case to be reported.

## Introduction

Atypical fibroxanthoma (AFX) is a rare neoplasm that is composed of fusiform, fibro-histiocytic pleomorphic spindle cells. It is usually considered a superficial and less aggressive variant of pleomorphic dermal sarcoma (PDS) or undifferentiated pleomorphic sarcoma (UPS) [1]. AFX usually occurs on sun-exposed areas and presents as a small pink-red, firm, asymptomatic solitary nodule. Most cases of AFX are locally aggressive and carry a good prognosis but in rare cases, reoccurrence and metastasis can occur [2]. We present a distinctive case of moderately differentiated squamous cell carcinoma (SCC) and AFX occurring as a collision tumor of the right shoulder. To our knowledge, this is the first case of collision tumor of SCC and AFX reported in the literature.

## Case presentation

A 77-year-old female with a past medical history of diabetes mellitus (DM), hyperlipidemia, hypertension (HTN), and seronegative rheumatoid arthritis presented with a pigmented skin lesion located on her right upper back. The patient first noticed the lesion three months earlier because it became irritated and painful. The lesion consisted of a 1.2 x 1.1 cm hyperpigmented, very well-circumscribed nodule, that was pink-red in color, irregular, moderately irritated, and firm in consistency with focal ulceration. A shave biopsy was performed and sent for histopathological examination.

**Figure 1:**
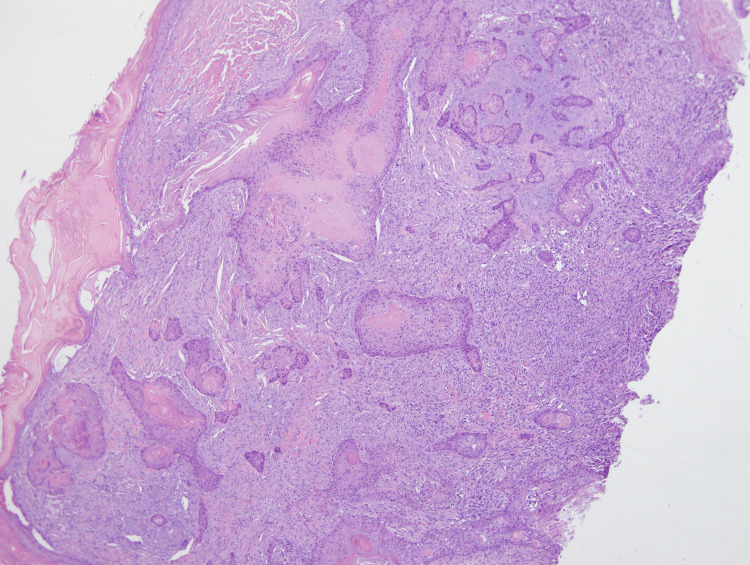
Medium power view clearly showing nests of SCC (center) surrounded by AFX. (4x) Magnification. SCC: squamous cell carcinoma; AFX: atypical fibroxanthoma

**Figure 2:**
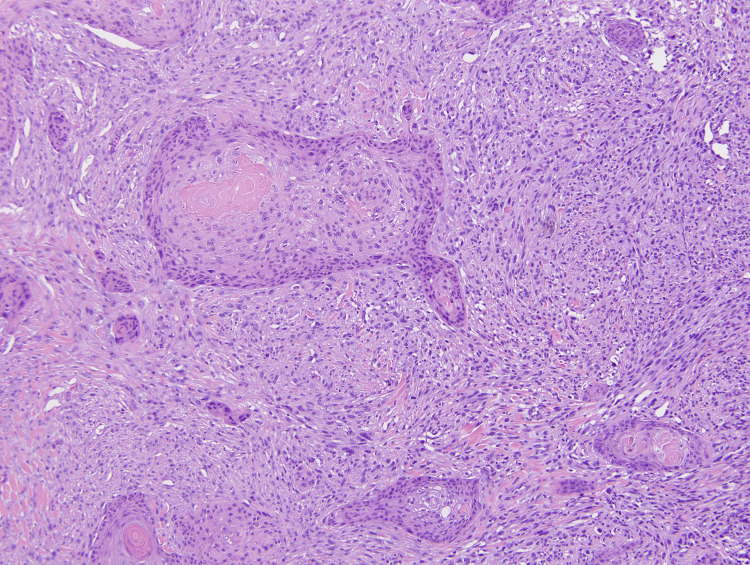
: Medium power view illustrating the presence of spindled to epithelioid, pleomorphic cells representing AFX and the ISCC component. (10x) Magnification. AFX: atypical fibroxanthoma; ISCC: invasive squamous cell carcinoma

Microscopic examination revealed an epidermal and dermal-based single lesion that had two types of cell population with distinct histopathological features. The aggregates of atypical keratinocytes with eosinophilic, keratinizing cytoplasm extending from the epidermis to deep down into the dermis were evident on hematoxylin and eosin (H&E) stain (Figures 1, 2). This moderately differentiated invasive SCC (ISCC) had a depth of invasion up to 3 mm (Figures 1, 2). No perineural or vascular invasion was identified, and all the resection margins were negative for ISCC.

**Figure 3:**
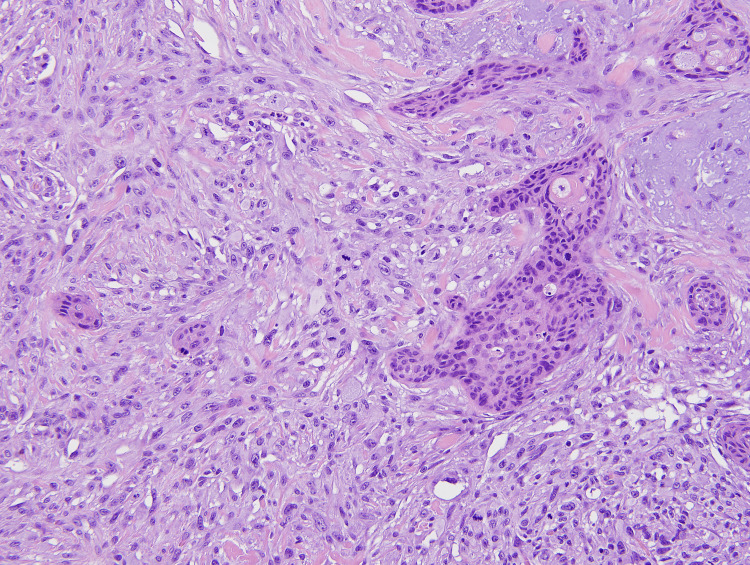
Spindle cells are arranged in irregularly sheets and fascicles with intermixed ISCC. (20x) Magnification. ISCC: invasive squamous cell carcinoma

**Figure 4:**
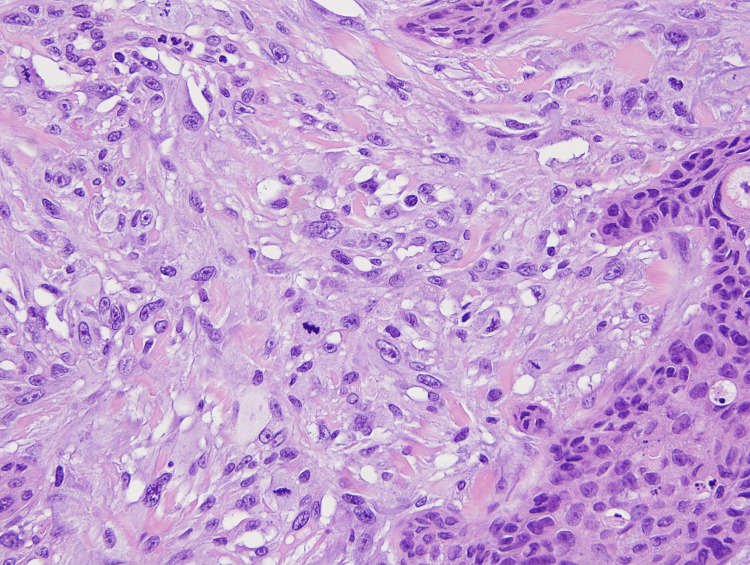
High power view illustrating the pleomorphic, multinucleated cells with atypical mitotic figures. (40x) Magnification.

Within the dermis and subcutis and between the nests of SCC, a highly atypical epithelioid to spindle cell neoplasm was present (Figures 1, 2). These spindle cells were arranged in irregular sheets and fascicles, mostly confined to the dermis (Figure 3). On high power, these cells appeared pleomorphic with abundant eosinophilic cytoplasm, occasional multinucleation, and numerous atypical mitotic figures with prominent nucleoli (Figure 4).

**Figure 5:**
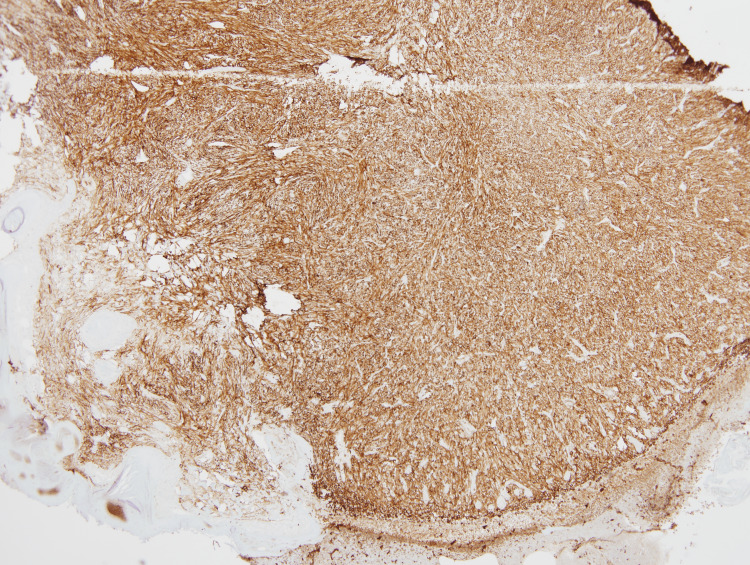
Immunohistochemistry showing diffuse expression of CD10 in AFX. (4x) Magnification. AFX: atypical fibroxanthoma

**Figure 6:**
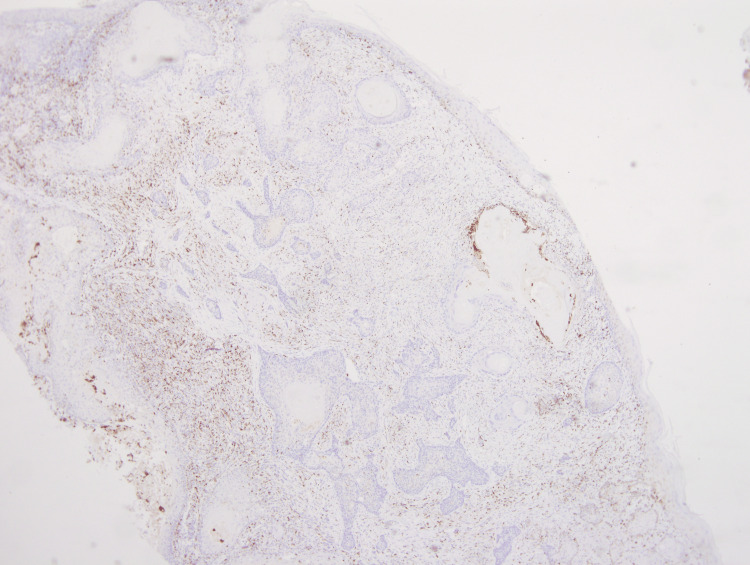
Immunohistochemistry showing diffuse expression of CD68 in AFX. The areas that are not staining brown represent the SCC. (4x) Magnification. AFX: atypical fibroxanthoma; SCC: squamous cell carcinoma

**Figure 7:**
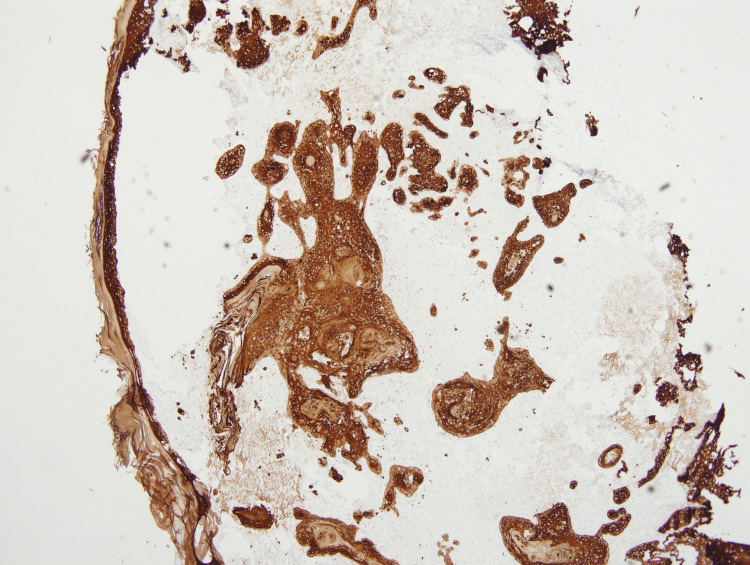
Immunohistochemistry, strong, diffuse expression of high molecular weight cytokeratin (HWMCK), CK5/CK6 in ISCC. (4x) Magnification. ISCC: invasive squamous cell carcinoma

**Figure 8:**
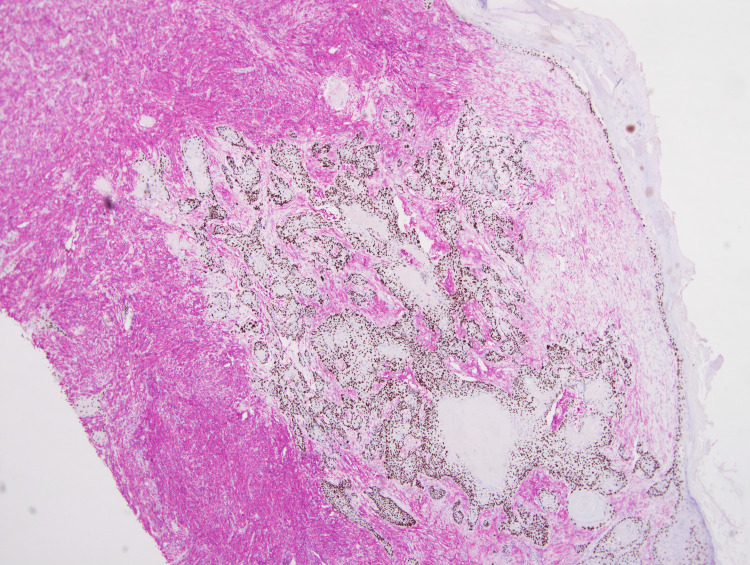
Dual immunohistochemistry, nuclear stain for p63 (brown) and red stain for CD10. (4x) Magnification.

These atypical spindle cells were diffusely positive for CD10 and CD68 (Figures 5, 6). The SCC showed diffuse and strong positive expression of high molecular weight cytokeratin (HMWCK) CK5/CK6 (Figure 7), and pan-cytokeratin. Figure 8 shows a combined IHC nuclear stain for p63 (red) and brown stain for CD10.

AFX measuring up to 8 mm in size in the greatest dimension with a maximum depth of invasion of 4.5 mm was seen. No necrosis or vascular or perineural invasion was identified. The resection margins were negative for AFX. 

## Discussion

This is an extremely rare case of collision tumor of moderately differentiated ISCC and AFX presenting as a right scapular nodular lesion. This is unprecedented in the English literature and the first such case to be reported. 

SCC and AFX are two separate dermal lesions that can present as pink-red, nodular, ulcerated lesions and clinically mimic each other. However, it is exceedingly rare to find these two distinct entities coexisting as one tumor. If they do coexist, then the tumor is called a collision tumor [1]. AFX is a primary mesenchymal uncommon dermal fibrohistiocytic tumor that mostly occurs in sun-exposed areas of skin. It is composed of fusiform, spindle, and epithelioid cells that are small to medium in size, hyperchromatic, and pleomorphic with high nuclear to cytoplasm ratio and mitotic activity [3]. It encompasses up to 0.2% of all skin tumors [4].

AFX was first described by Helwig in 1960 and was considered a neoplastic process of fibrohistiocytic origin [5]. The term "atypical fibroxanthoma" suggests that the tumor configuration is primarily composed of xanthomatous type of cells with a variable quantity of fibrohistiocytic cells that frequently exhibit significant cellular atypia [6]. AFX is commonly considered and classified as a benign lesion with a good prognosis, but in rare cases, recurrence and metastasis can happen [7]. It has been proposed that neoplastic lesions with adverse histopathological traits and the lesions that extend deep into subcutaneous tissues and muscles are more aggressive and carry a high potential for recurrence and metastasis. These lesions should be categorized as pleomorphic dermal sarcomas (PDS) instead of AFX [6].

Sunlight exposure including ultraviolet radiation is a significant risk factor for the development of many skin cancers including ISCC and AFX [8]. Ultraviolet radiations also have effects on DNA repair mechanisms and tumor suppressor genes like P53 and promote tumor genesis in many skin cancers. Some patient populations at risk include immunocompromised patients, organ transplant patients, and patients with a previous history of chemotherapy and radiation therapy [9].

## Conclusions

Histologically and clinically, ISCC and AFX can mimic a wide range of different dermal lesions. Differentiating and arriving at the correct diagnosis of these lesions is necessary and can be challenging for clinicians and pathologists. Accurate diagnosis is very important because it plays a crucial role in therapeutic options and prognostic factors of these lesions. The purpose of presenting this unique case of collision tumor is to provide awareness of the existence of this entity to pathologists and clinicians.
